# Psychological influences on distance estimation in a virtual reality environment

**DOI:** 10.3389/fnhum.2013.00580

**Published:** 2013-09-18

**Authors:** Kohske Takahashi, Tobias Meilinger, Katsumi Watanabe, Heinrich H. Bülthoff

**Affiliations:** ^1^Research Center for Advanced Science and Technology, The University of TokyoTokyo, Japan; ^2^Department of Human Perception, Cognition and Action, Max Planck Institute for Biological CyberneticsTübingen, Germany; ^3^Department of Brain and Cognitive Engineering, Korea UniversitySeoul, Korea

**Keywords:** distance perception, spatial perception, virtual reality environment, personal space, object geometry

## Abstract

Studies of embodied perception have revealed that social, psychological, and physiological factors influence space perception. While many of these influences were observed with real or highly realistic stimuli, the present work showed that even the orientation of abstract geometric objects in a non-realistic virtual environment could influence distance perception. Observers wore a head mounted display and watched virtual cones moving within an invisible cube for 5 s with their head movement recorded. Subsequently, the observers estimated the distance to the cones or evaluated their friendliness. The cones either faced the observer, a target behind the cones, or were oriented randomly. The average viewing distance to the cones varied between 1.2 and 2.0 m. At a viewing distance of 1.6 m, the observers perceived the cones facing them as closer than the cones facing a target in the opposite direction, or those oriented randomly. Furthermore, irrespective of the viewing distance, observers moved their head away from the cones more strongly and evaluated the cones as less friendly when the cones faced the observers. Similar distance estimation results were obtained with a 3-dimensional projection onto a large screen, although the effective viewing distances were farther away. These results suggest that factors other than physical distance influenced distance perception even with non-realistic geometric objects in a virtual environment. Furthermore, the distance perception modulation was accompanied by changes in subjective impression and avoidance movement. We propose that cones facing an observer are perceived as socially discomforting or threatening, and potentially violate an observer's personal space, which might influence the perceived distance of cones.

## Introduction

Perceived space is not necessarily veridical, as demonstrated by many optical illusions (e.g., Müller-Lyer illusion, Ponzo illusion, and the Ebbinghaus illusion). Apart from illusions, spatial perception is susceptible to the influences of observer's psychological and physiological states. Hills appear steeper after a 1-h run (Proffitt et al., 1995; Proffitt, 2006), and a glass of water looks larger when observers feel thirsty (Veltkamp et al., 2008). These studies support the notion of embodied perception, according to which observers' mental and bodily states modify spatial perception (Proffitt, 2006).

Of our particular interest is distance perception. Distance modulates, explicitly and implicitly, the way we behave in the real world (e.g., personal space, Liberman et al., 2007). Recently, many studies have examined how factors other than physical distance influence distance perception. For example, desired objects are felt as nearer or are seen as closer (Balcetis and Dunning, 2010; Alter and Balcetis, 2011). Wearing a backpack or throwing a heavy ball results in larger subsequent distance estimations compared with wearing no backpack or throwing a light ball (Proffitt et al., 2003; Witt et al., 2004). Threatening objects (e.g., a living tarantula) are perceived as closer (Cole et al., 2012). A location related to a rival group (e.g., Fenway Park for a Yankees fan) is imagined as nearer when accompanied by a feeling of threat (Xiao and Van Bavel, 2012). These studies imply that distance perception reflects more than physical distance, namely, social, psychological, and physiological aspects.

Thus far, the influence of the social, psychological, or physiological factors on distance perception have been tested primarily in real world situations with semantically meaningful stimuli, in line with the notion of embodied perception (Proffitt, 2006). These situations evoke associations between the presented stimuli and expected reward or punishment (e.g., a tarantula might hurt us at a closer distance). It would be plausible to argue that the expectations of reward or punishment (i.e., prospect and threat) influence distance perception by modulating psychological states. In the present study, we simplified the situation so that visual stimuli no longer afforded realistic rewards or punishments and observers were aware that the affective values associated with the visual stimuli, if any, were not real. For this purpose, we investigated the modulation of distance perception using a virtual environment and meaningless geometric objects. A virtual environment is an experimental tool used increasingly in a wide range of contexts from navigation behavior (e.g., Frankenstein et al., 2012) to social phenomena (e.g., personal space, Bailenson et al., 2003). In virtual environments, objects are typically not real, which enables us to examine situations where observers know that the objects are *not* associated with realistic rewards or punishments (e.g., a tarantula in a virtual environment will not hurt us even at the closest distance). We presented cone-shaped objects and manipulated the orientation of the cones. The tips of the cones faced an observer, faced another location in a virtual environment, or were oriented randomly. We expected the psychological reaction to vary depending on cone orientation. In particular, the cone tips that faced the observer might induce threat or unfriendliness as in the real world situations, wherein some people develop aichmophobia, an excessive fear of sharp or pointy objects such as needles (Morse and Cohen, 1983; Shabani and Fisher, 2006).

## Experiment 1

### Materials and methods

#### Observers

Fourteen paid volunteers (3 females, age 19–53 years) participated in the experiment after giving written informed consent. The experimental setup was approved by the local ethics committee.

#### Apparatus

During the experiment, the observers stood behind a horizontal bar and grabbed a gamepad that was attached to the bar. We controlled stimulus generation and data acquisition using MATLAB with the Psychtoolbox extension (Brainard, 1997; Pelli, 1997). Visual stimuli were presented through a stereoscopic head mounted display (Kaiser SR80) with a field of view of 63° (horizontal) × 53° (vertical), a resolution of 1280 × 1024 pixels for each eye, with 100% overlap, and a 60 Hz refresh rate. We fixed the inter-pupil distance for the stereo projection at 6 cm for all observers. The observers' head movements (i.e., translation and rotation) were monitored by four high-speed motion capture cameras (Vicon® MX 13) with a 120 Hz sampling rate; they were used for online stereo projection and offline head movement analysis. The stereo presentation setup allowed the observers to feel immersed in the virtual environment.

#### Stimuli

**Figure 1:**
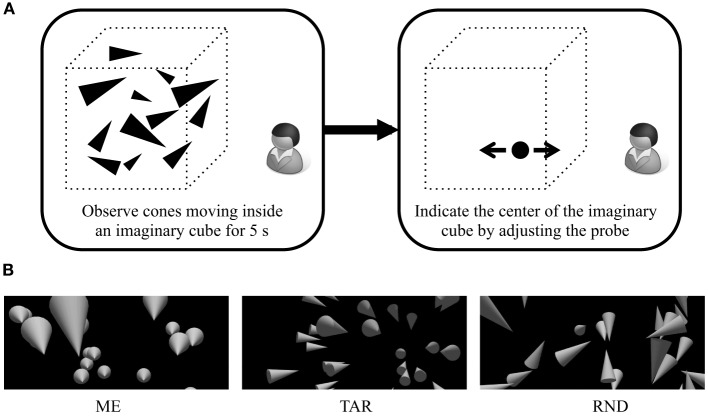
**(A)** A schematic illustration of the experiment. The participants viewed virtual cones moving inside an imaginary cube for 5 s, after which they indicated the center of the imaginary cube. **(B)** Example snapshots of a visual image of the ME (all cones are facing the observer), TAR (all cones are facing an invisible target located behind the cones), and RND (the cone orientations are random) conditions.

The visual stimulus consisted of 50 cone-shaped 3-dimensional (3-D) objects (Figure 1). The cones were of 7 cm radius and 30 cm height and moved inside an imaginary cube (200 × 200 × 200 cm) located at the observer's eye height. The mean viewing distance, from the observer to the center of the imaginary cube, was 120 cm (the cone distances ranged from 20 to 220 cm), 160 cm (the cone distances ranged from 60 to 260 cm), or 200 cm (the cone distances ranged from 100 to 300 cm). The cones moved at 75 cm/s in a direction randomly determined for each cone; each cone's direction changed every 333 ms. When the center of the cone's mass (i.e., three quarters of the middle line down from the vertex) reached a cube wall, it was reflected from the wall. The cones had a direction (i.e., based on the orientation of their tips, Figure 1B). In the ME condition, the cones were pointing toward the observer's chest. In the TAR condition, the cones were pointing toward an invisible target placed 340 cm away from the observer. In the RND condition, the cones were pointing in pseudo-random directions. The cone directions in the RND condition were determined based on an algorithm similar to that used by Gao et al. (2010). The tip of a cone directed off a virtual line from the center of the cone to the center of the imaginary cube by a specific degree ranging from −90° to 90°. The deviation amount was fixed for each cone and was randomly determined. This made the motion profile of each cone as similar as possible for the different cone direction conditions.

#### Procedure

A button press started the trial. After viewing a blank screen for 0.5 s, the observer viewed the moving cones for 5 s. After the visual stimuli disappeared, a probe circle appeared at a random distance (110–210 cm away) from the observer. The observer indicated the center of the imaginary cube by moving the probe along an invisible line, which was extended horizontally through the middle of the imaginary cube, and pressing a button (Figure 1A).

We used a 3 × 3 within-subjects design. The factors were viewing distance (3 levels: 120, 160, and 200 cm), and cone direction (3 levels: ME, TAR, and RND). Each condition was repeated six times resulting in 54 trials, which were presented in a random order. Before the experiment, the observers were allowed to practice as long as they wanted.

### Results

After removing values that deviated greater than 3 standard deviations from the overall mean computed for all observations (i.e., the outlier observations), the data were submitted to a mixed model analysis with the within-subjects factors of viewing distance and cone direction. We also reported partial eta squared (η^2^_*p*_) values derived from the data aggregated for each observer and for each of the conditions.

#### Distance estimations

**Figure 2:**
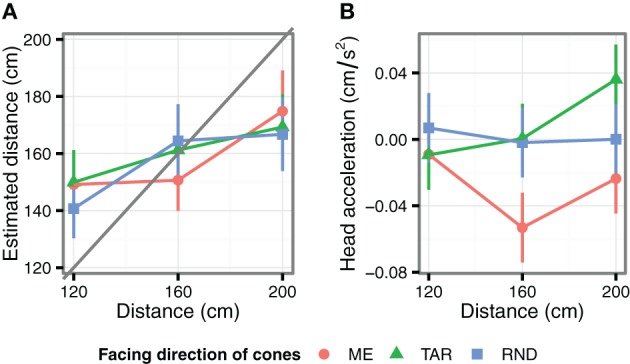
**Results of Experiment 1. (A)** The average estimated distance as a function of viewing distance and cone direction. **(B)** The average head acceleration along the depth axis during the stimulus observation period. Positive values indicate accelerations toward the cones. The error bars indicate the standard error of the mean.

Figure 2A shows the distance estimation results. As expected, the estimated distances differed depending on the viewing distance [*F*_(2, 708)_ = 40.0, *p* < 0.001, η^2^_*p*_ = 0.67]. Thus, the observers could distinguish between the different viewing distances. Note the slopes were shallower than the veridical estimation^1^. That is, the observers underestimated the distance at 200 cm [*F*_(1, 13)_ = 4.99, *p* = 0.044, η^2^_*p*_ = 0.28], and overestimated the distance at 120 cm [*F*_(1, 13)_ = 5.95, *p* = 0.030, η^2^_*p*_ = 0.31]. No deviation from the actual distance was found in the 160 cm condition (*F* < 1).

The cone directions did not significantly bias the distance estimations [*F*_(2, 708)_ = 0.61, *p* = 0.544, η^2^_*p*_ = 0.07]. However, we found a significant interaction between viewing distance and cone direction [*F*_(4, 708)_ = 3.38, *p* = 0.009, η^2^_*p*_ = 0.26]. At the 160 cm viewing distance, the estimated distance was significantly shorter in the ME condition compared to the TAR condition [*F*_(1, 151)_ = 4.37, *p* = 0.038, η^2^_*p*_ = 0.28] and the RND condition [*F*_(1, 149)_ = 6.72, *p* = 0.010, η^2^_*p*_ = 0.23]. On the other hand, at 120 cm, the estimated distances in the RND condition were significantly shorter than in the TAR condition [*F*_(1, 150)_ = 6, *p* = 0.016, η^2^_*p*_ = 0.63] and tended to be shorter than in the ME condition [*F*_(1, 149)_ = 3.81, *p* = 0.053, η^2^_*p*_ = 0.22]^2^. At the viewing distance of 200 cm, we did not observe an effect on cone direction (*F* < 1).

#### Head movements

We examined head movements along the depth axis. Figure 2B shows the observers' head accelerations in each of the conditions, averaged over 5 s. The head acceleration differed significantly between the cone direction conditions [*F*_(2, 700.2)_ = 3.78, *p* = 0.023, η^2^_*p*_ = 0.28], irrespective of viewing distance (i.e., no interaction between viewing distance and cone direction, [*F*_(4, 700.4)_ = 1.14, *p* = 0.336, η^2^_*p*_ = 0.10]. In the ME condition, the observers accelerated their heads more strongly away from the cones than in the TAR condition [*F*_(1, 462.4)_ = 6.43, *p* = 0.012, η^2^_*p*_ = 0.41], or in the RND condition [*F*_(1, 463.2)_ = 4.19, *p* = 0.041, η^2^_*p*_ = 0.33]. For the velocity of the head movements, the effect of viewing distance, cone direction, and the interaction were not statistically significant (*F*s < 1.32, *ps* > 0.26).

### Discussion

The results of Experiment 1 suggested that the cone direction modulated the distance estimations; this modulation effect depended on the viewing distance. The cones facing toward the observers were perceived as closer when they appeared in the viewing distance of 160 cm. Furthermore, the observers moved away from the cones more strongly when the cones faced them, which implies avoidance behavior. A post-experiment questionnaire also suggested that the observers experienced the cones facing them as more negative (less friendly or more threatening) than those facing the other directions. Thus, the modulation of the distance estimations might be related to the observers' negative impressions of the cones. In Experiment 2, therefore, we directly tested whether cone direction affected the subjective impression of the cones.

## Experiment 2

### Materials and methods

Nine paid volunteers (2 females, age 19–24 years) participated after giving a written informed consent. The material and methods were identical to those of Experiment 1 except for the following. The observers sat on a chair and wore a different head mount display (Sony HMZ-T2). Their head movement was not monitored. After the 5-s stimulus presentation, the observers rated the “friendliness” of the cones on a 7-point scale with the poles labeled as “hostile” and “friendly” by mouse clicking.

### Results

**Figure 3:**
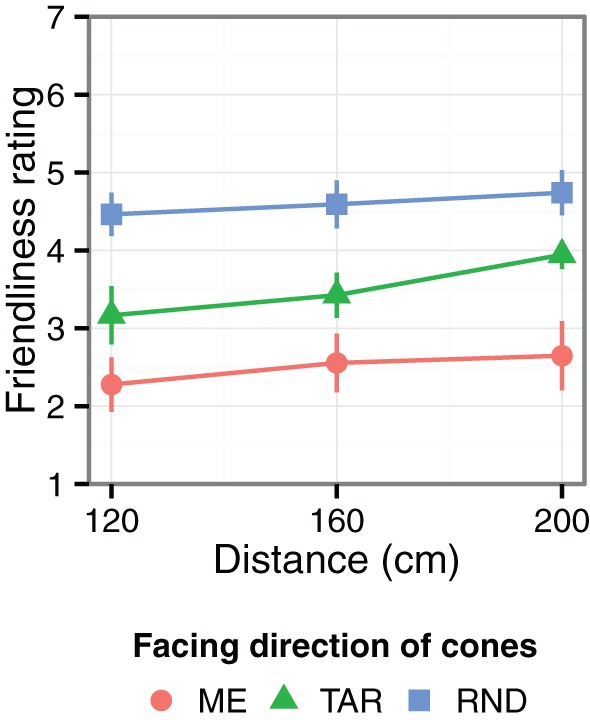
**The results of Experiment 2.** Larger values indicate that the cones were evaluated as being friendlier and less hostile. The error bars indicate the standard error of the mean.

Figure 3 shows the results of Experiment 2. The cone direction affected the friendliness ratings of the cones [*F*_(2, 469)_ = 88.0, *p* = 0.001, η^2^_*p*_ = 0.62]. The cones were rated as less friendly (or more hostile) in the ME condition than in the TAR condition [*F*_(1, 310)_ = 49.7, *p* < 0.001, η^2^_*p*_ = 0.49], which were in turn rated as less friendly than in the RND condition [*F*_(1, 310)_ = 45.2, *p* < 0.001, η^2^_*p*_ = 0.58]. In addition, viewing distance significantly modulated the friendliness ratings [*F*_(2, 496)_ = 4.49, *p* = 0.012, η^2^_*p*_ = 0.28], which increased linearly with the viewing distance [*F*_(1, 472)_ = 9.02, *p* = 0.003, η^2^_*p*_ = 0.31]. The interaction between viewing distance and cone direction was not statistically significant (*F* < 1).

### Discussion

The cones were rated as friendlier when they were facing the target rather than the observer, and when they were further away. The effect of distance estimations depended on viewing distance. In contrast, the effect of cone direction on the friendliness ratings did not depend on viewing distance. Although the decreased perceived friendliness of the cones that faced the observer is one factor that affected the distance estimations, other factors affected the pattern of distance estimates.

## Experiment 3

First, we tested whether the observed effects were specific to a virtual reality setup using a head mounted display which allowed head movements. To do so, we used a projection screen with shutter glasses to emulate 3-D vision with the head fixed. Second, we wanted to clearly identify the distance at which the effect was observed. Therefore, we concentrated on the distance difference between the ME and TAR conditions and examined the effect of cone direction at seven different viewing distances. Last, we wanted to determine the within-participant relationship between the distance estimates and the friendliness ratings.

### Materials and methods

Nineteen paid volunteers (12 females, age 19–27 years) participated after giving a written informed consent. The visual stimuli were presented on a large screen by a 3D stereo projector (Sight 3D, Solidray Co. Ltd.) and stereo shutter glasses (3D Vision, NVIDIA). The refresh rate of the projection was 120 Hz (i.e., 60 Hz for each eye). The screen was 133.5 cm (height) × 178 cm (width). The height of the center of the screen above the ground was 144 cm. The observers sat on a chair in front of the screen with their head fixed on a chin rest. The distance from the screen to the observer's eye position was 213.5 cm. The visual stimuli were identical to those used in Experiment 1 except for the following. Since the visual angle (field of view) was smaller than that in Experiment 1, the size of the imaginary cube was 100 × 100 × 100 cm and the number of cones was 25. In the ME and TAR conditions, we used 7 different distances from 100 to 220 cm at 20 cm intervals. We omitted the RND condition. The observers first engaged in the distance estimation procedure as in Experiment 1. Next, they rated the cones' friendliness as in Experiment 2. For the distance estimations, each stimulus was repeated six times. Hence, there were 84 trials. For the subjective ratings, each stimulus was presented once.

### Results

#### Distance estimations

**Figure 4:**
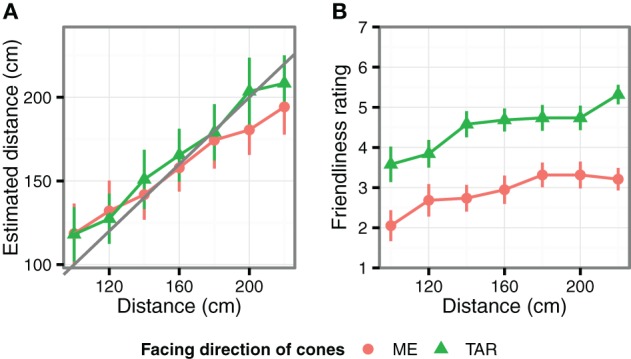
**The results of Experiment 3. (A)** The average estimated distances as a function of viewing distance and cone direction. **(B)** The average rating scores. The error bars indicate standard error of the mean.

The results are shown in Figure 4A. We found statistically significant effects for cone direction [*F*_(1, 1532)_ = 16.2, *p* < 0.001, η^2^_*p*_ = 0.33], and for viewing distance [*F*_(6, 1532)_ = 2.7, *p* < 0.001, η^2^_*p*_ = 0.83]. There was a significant interaction effect found between cone direction and viewing distance [*F*_(6, 1532)_ = 2.41, *p* = 0.025, η^2^_*p*_ = 0.15]. The distance estimations were significantly closer in the ME condition compared to the TAR condition only at the 200 cm and 220 cm viewing distances (*F*s > 15.0, *ps* < 0.001). No differences were found at the other viewing distances (*F*s < 1.95, *ps* > 0.165). Notably, the distance estimations were more veridical in Experiment 3 than in Experiment 1. We found no significant differences between the presented distances and the estimated distances at any of the viewing distances (*F*s < 1.79, *ps* > 0.198).

#### Friendliness ratings

Figure 4B shows the average score of the friendliness ratings. The results were generally consistent with those of Experiment 2. The cones in the ME condition were rated as less friendly than the cones in the TAR condition [*F*_(1, 234)_ = 84.9, *p* < 0.001, η^2^_*p*_ = 0.51]. The effect of distance was also significant [*F*_(1, 234)_ = 4.78, *p* < 0.001, η^2^_*p*_ = 0.24]. The friendliness ratings almost monotonically increased with the viewing distance. The viewing distance by cone direction interaction was not statistically significant (*F* < 1). Furthermore, the overall within-participant correlation between the distance estimations and the friendliness ratings was statistically significant (*r* = 0.13, *p* = 0.036). The positive correlation observed supports the relation between these two measures.

### Discussion

The results of Experiments 1 and 2 were at least partially replicated in Experiment 3, which used a different virtual reality presentation method. Consistent with Experiment 2, the cones facing the observer were rated as less friendly, irrespective of the viewing distance. Furthermore, the cone direction modulated the distance estimates; similar to the results of Experiment 1, this modulation depended on the viewing distance. We compared the results of Experiments 1 and 3. In both experiments, the cones facing toward the observers were perceived as closer. This effect depended on the viewing distances, and only the effective viewing distance was different between Experiments 1 and 3. This point is further addressed in the General Discussion section.

## General discussion

The present study examined whether factors other than physical distance—the orientation of simple geometric objects—influenced distance perception in a virtual environment. In Experiment 1, the observers estimated the virtual cones facing them as closer than the cones facing other directions, when the cones were presented at a certain distance (i.e., 60–260 cm away). Furthermore, the cone direction affected the observers' head movements (Experiment 1) and the subjective impression of the friendliness of the cones (Experiment 2). When the tips of the cones faced the observers, the observers moved away from the cones and rated the cones as less friendly (more hostile). These effects were observed irrespective of the viewing distance. Experiment 3 replicated the distance estimation and friendliness rating results, although the effective viewing distance was further away (greater than 150–270 cm).

The effect on distance perception could not be a direct effect of geometric factors. As a cone is a 3-D object, the position of the cone is somewhat ambiguous when referred to by a point-shaped probe. For example, if the tips of a cone served as a representative point, the cones facing toward the observers would be estimated as closer than the cones facing the opposite direction, when the center of the cones was located at the same position, as was the case in the present experiment. If these geometric factors played a role in the modulation of distance perception, then cone direction would have influenced distance perception irrespective of viewing distance. We found, however, that the modulation of distance perception due to the cone direction depended on the viewing distance, which is not consistent with an account based on a direct effect of geometric factors. Rather, the geometric factor—whether cones faced toward the observers or not—would affect the distance perception through mediating psychological factors, such as experienced unfriendliness or perception of a threat.

In Experiments 1 and 3, the observers perceived the cones facing toward them as closer, when the cones were presented at certain viewing distances. The effective viewing distances were, however, not the same; they were from 60 to 250 cm and 150 to 270 cm in Experiments 1 and 3, respectively. The visual stimuli differences—the size of the imaginary cube and the number of cones—might have caused the difference in the effective viewing distances. However, at the moment, we speculate that differences in the devices used for the 3-D stereo presentation were responsible for the dependency on the different viewing distance. Distance in virtual environments is not necessarily veridical, but sometimes distorted compared with real spaces (Loomis and Knapp, 2003; Thompson et al., 2004). The amount of distortion depends on the setup used. The lower slope of the estimated distance against the presented distance in Experiment 1 (Figure 2A) suggests that the presented distances might be mapped onto a smaller subjective range. This was less of a factor in Experiment 3, in which the presented and estimated distances matched more closely (Figure 4A). Consequently, the subjective ranges within which cone orientation influenced distance perception might have been even more similar than suggested by the presented distances.

Several psychological factors are known to influence distance perception. Many studies have examined such influences in real world situations with meaningful stimuli (Proffitt et al., 2003; Witt et al., 2004; Balcetis and Dunning, 2010; Alter and Balcetis, 2011; Cole et al., 2012; Xiao and Van Bavel, 2012). In contrast, the aim of the present study was to examine distance perception in a virtual environment with simple visual stimuli. The virtual cones could not physically hurt the observers (and the observers knew this); nevertheless, the observers perceived the virtual cones facing toward them as closer when they were presented at a specific viewing distance. Moreover, distance perception modulation was accompanied by observers' avoidance behavior and negative subjective impression of the cones (i.e., they were rated as less friendly or more hostile). Thus, distance perception modulation was observed even when the observers were aware that the reward or punishment was virtual.

The cones facing the observers at a specific viewing distance were perceived as closer. Perhaps the modulation of distance perception was mediated by the perception of an emotional threat and/or social discomfort. One possibility is related to the fact that pointy objects tend to evoke aversion (Morse and Cohen, 1983; Shabani and Fisher, 2006); the aversion evoked might have been stronger when the cone tips faced the observers. This fits with the avoidance behavior indicated by the head tracking data as well as the less friendly ratings. The cones might also trigger social processing related to the regulation of interpersonal distance. The observers' backwards movements when faced by the cone tips could be also considered as the signature of implicit avoidance behavior. This is consistent with the finding that observers in a virtual environment keep the larger distance with a virtual avatar facing toward them (Bailenson et al., 2003). Previous studies suggested that socially or emotionally negative targets in the real world were felt or perceived as closer (Cole et al., 2012; Xiao and Van Bavel, 2012). Another possibility to explain the effect of the cones' direction on the distance estimations is that the cones facing toward the observers might be perceived as potentially approaching them. Many studies suggested that approaching objects lead to specific (negative in most cases) perceptual and social states (Mühlberger et al., 2008; Tajadura-Jiménez et al., 2010).

Although the cones facing toward the observers were rated as less friendly regardless of the viewing distance, they were perceived as being closer at specific viewing distances. Therefore, even if perceived friendliness was related to the modulation of distance estimation, it would not be a direct cause of the modulation. The distance perception modulation might be related to the violation of personal space (Liberman et al., 2007). Wilcox et al. (2006) showed that objects in a virtual environment were felt as intrusive, when the viewing distance was less than 100 cm. The modulation of distance perception by the cones' direction might take place only when they appear near the intrusiveness boundary (i.e., the personal space boundary). Objects much closer than the boundary would be perceived as violating personal space, irrespective of their perceived friendliness, while objects far away from the boundary would be perceived as not violating personal space. On the other hand, when the objects were close to the boundary, the perception of them as intrusive and violating personal space might depend on their friendliness. At this specific viewing distance, the cones facing toward the observers that resulted in the negative reactions (i.e., avoidance behavior and less friendly ratings) might be felt as intrusive and violating; if they were facing another direction, they might be perceived as non-intrusive. A recent study demonstrated that the representation of personal space is sensitive to social factors (Teneggi et al., 2013). According to this view, the intrusive cones would be perceived as closer since they violated the observers' personal space (Schnall, 2011). Although these accounts are speculative, they warrant further investigation by combining a virtual environment, personal space, and distance perception.

In sum, the present study showed that the orientation of simple geometric objects in a virtual environment could influence their perceived distance from observers, their perceived friendliness, and implicit avoidance behavior. Several issues concerning distance perception in virtual environments remain open. For example, a direct comparison of distance perception using the same stimuli in a real and a virtual environment would help us understand how the disconnection from the real world (real rewards and punishments) affect our distance perception. The results of the present study suggest that the perception of an object as closer and having a negative impression about that object co-occur. In contrast, in the real world, we form positive and negative impressions of objects that are perceived as closer. For instance, desired objects are perceived as closer (Balcetis and Dunning, 2010). How virtual rewards affect distance perception, warrants further investigation. Our study also implies that distance estimation may serve as an objective measure for the strength of psychological reactions in the social domain in virtual environments. There has been an increase in the combination of social communication with virtual environments. Examining distance perception in virtual environments with an emphasis on psychological and social aspects will lead to the development and application of user-friendly technologies.

### Conflict of interest statement

The authors declare that the research was conducted in the absence of any commercial or financial relationships that could be construed as a potential conflict of interest.

## Footnotes

^1^Distance perception in virtual environments is known to be distorted relative to real world distance perception (Loomis and Knapp, 2003; Thompson et al., 2004). Therefore, we were not surprised to observe effects in this direction. However, this was beyond the focus of the present study and we will not speculate further about the potential reasons for these effects.

^2^It is unclear why the cones of random directions were perceived as closer at the short viewing distance. At the short distance, the cones of random directions might have appeared as more crowded and less organized. Consequently, they might have “overwhelmed” the observers to a greater extent than the cones of the more ordered conditions, and were thus perceived as closer. Because our primary interest was the effect of the cones facing toward the observers, we did not provide an in-depth discussion of the comparisons between the RND vs. the ME and the TAR conditions.
